# Inhibition of Melanogenesis by Gallic Acid: Possible Involvement of the PI3K/Akt, MEK/ERK and Wnt/β-Catenin Signaling Pathways in B16F10 Cells

**DOI:** 10.3390/ijms141020443

**Published:** 2013-10-14

**Authors:** Tzu-Rong Su, Jen-Jie Lin, Chi-Chu Tsai, Tsu-Kei Huang, Zih-Yan Yang, Ming-O Wu, Yu-Qing Zheng, Ching-Chyuan Su, Yu-Jen Wu

**Affiliations:** 1 Antai Medical Care Cooperation Antai Tian-Sheng Memorial Hospital, Pingtung 92842, Taiwan; E-Mails: a081002@mail.tsmh.org.tw (T.-R.S.); Swimmingteddy@gmail.com (T.-K.H.); a081001@mail.tsmh.org.tw (C.-C.S.); 2 Graduate Institute of Veterinary Medicine, National Pingtung University of Science and Technology, Pingtung 91202, Taiwan; E-Mail: q87634@yahoo.com.tw; 3 Kaohsiung District Agricultural Improvement Station, Pingtung 900, Taiwan; E-Mail: tsaicc@mail.kdais.gov.tw; 4 Graduate Institute of Food Science, National Pingtung University of Science and Technology, Pingtung 91202, Taiwan; E-Mail: gini0307@yahoo.com.tw; 5 Department of Beauty Science, Meiho University, Pingtung 91202, Taiwan; E-Mails: mm086966@yahoo.com.tw (M.-O.W.); yqch20@yahoo.com.tw (Y.-Q.Z.); 6 Graduate Institute of Applied Health and Biotechnology, Meiho University, Pingtung 91202, Taiwan

**Keywords:** gallic acid, melanogenesis, microphthalmia-associated transcription factor (MITF), tyrosinase, TRP1, Dct, MEK/ERK, PI3K/Akt, Wnt/β-catenin

## Abstract

Gallic acid is one of the major flavonoids found in plants. It acts as an antioxidant, and seems to have anti-inflammatory, anti-viral, and anti-cancer properties. In this study, we investigated the effects of gallic acid on melanogenesis, including the activation of melanogenesis signaling pathways. Gallic acid significantly inhibited both melanin synthesis and tyrosinase activity in a dose- and time-dependent manner, and decreased the expression of melanogenesis-related proteins, such as microphthalmia-associated transcription factor (MITF), tyrosinase, tyrosinase-related protein-1 (TRP1), and dopachrome tautomerase (Dct). In addition, gallic acid also acts by phosphorylating and activating melanogenesis inhibitory proteins such as Akt and mitogen-activated protein kinase (MEK)/extracellular signal-regulated kinase (ERK). Using inhibitors against PI3K/Akt (LY294002) or MEK/ERK-specific (PD98059), the hypopigmentation effect was suppressed, and the gallic acid-initiated activation of MEK/ERK and PI3K/Akt was also revoked. Gallic acid also increased GSK3β and p-β-catenin expression but down-regulated p-GSK3β. Moreover, GSK3β-specific inhibitor (SB216763) restored gallic acid-induced melanin reduction. These results suggest that activation of the MEK/ERK, PI3K/Akt, and inhibition of Wnt/β-catenin signaling pathways is involved in the melanogenesis signaling cascade, and that activation by gallic acid reduces melanin synthesis via down-regulation of MITF and its downstream signaling pathway. In conclusion, gallic acid may be a potentially agent for the treatment of certain skin conditions.

## 1. Introduction

Melanocytes are located in the basal layer of the epidermis and are responsible for producing melanin, a substance that gives skin and hair their pigments. Melanin is synthesized in melanoctyes and transported to the surrounding epidermal keratinocytes to protect cells against the damaging effects of UV radiation [1]. However, abnormal melanogenesis causes pigmentary disorders, including medical conditions such as hypopigmentation (vitiligo and albinism) or hyperpigmentation (solar lentigo, chlosma, and freckles) [2]. The expression and activation of tyrosinase is important in the control of melanogenesis, since it acts as the catalyst in the rate limiting reaction of the melanogenic pathway [3]. Therefore, inhibitors of tyrosinase have been used in skin-whitening and cosmetic products.

On the other hand, melanin synthesis is activated by several signal transduction pathways, including the cAMP-mediated pathway, which plays an important role in melanogenesis regulation [4]. UV radiation stimulates adenyl cyclase to increase the cAMP level, which in turn binds to the Gs-protein-coupled receptor melanocortin receptor 1 (MC1R) in the human epidermis, and then activates cAMP-dependent protein kinase A (PKA) and other regulatory proteins [4]. PKA subsequently phosphorylates the cAMP response element-binding protein (CREB), which is known to be an activator of microphthalmia-associated transcription factor (MITF) gene expression [5,6]. Initiation of CREB phosphorylation has been shown to induce MITF transcription, a transcription factor for all melanogenic proteins, which up-regulates the expression of tyrosinase, tyrosinase-related protein-1 (TRP-1), and dopachrome tautomerase (Dct), consequently increasing melanin synthesis [7]. Furthermore, a 90-kDa ribosomal S6 kinase (RSK-1), the downstream kinase of ERK (extracellular signal-regulated kinase), has been demonstrated to phosphorylate MITF. The phosphorylated MITF decreases in stability, eventually leading to its degradation [8].

Many studies have been performed to explain the specific mechanism that regulates melanin synthesis. Recent studies have suggested that the MEK-ERK kinase cascade and the phosphatidylinositol 3-kinase (PI3K)/Akt pathway are involved in the regulation of melanin synthesis [9]. Activation of MEK/ERK and PI3K/Akt phosphorylates MITF, which promotes its degradation, therefore leading to inhibition of melanin production [10,11].

It is known that the Wnt signaling pathway is involved in MITF expression. Activation of Wnt pathway inhibits phosphorylation of glycogen synthase kinase 3β (GSk3β) and leads to β-catenin accumulation [12–14]. The accumulated β-catenin is transported into the nucleus and form a complex with the lymphoid-enhancing factor/T-cell factor (LEF/TCF) transcription factor, after which MITF expression is increased [15]. In addition, GSk3β is implicated to regulate melanogenesis. Inhibition of GSk3β activity could increase melanine synthesis through Wnt/β-catenin pathway activation [16]. Therefore, activation of Wnt/β-catenin signaling pathway may increase melanin production.

Many widely used lightening compounds, such as arbutin or kojic acid, are isolated from botanical extracts or natural sources. Gallic acid (3,4,5-trihydroxybenzoic acid), a naturally occurring plant polyphenol antioxidant, extracted from oak bark, gallnuts, witch hazel, red wines, tea, and other natural plants [17–21], has been shown to have various biological properties, including antioxidant, anticancer, and anti-inflammatory activities [22,23]. Previous reports have suggested that gallic acid exhibits antimelanogenic activity in addition to antioxidant properties by suppressing reactive species generation and maintaining a higher reduced glutathione to oxidized glutathione ratio (GSH/GSSG) [24]. However, the effects of gallic acid on the melanogenesis mechanism are not fully understood. In this study, we investigated the effects of gallic acid on melanogenesis and its molecular mechanism by using an *in vitro* model of B16F10 melanocyte cells.

## 2. Results

### 2.1. Effects of Gallic Acid on Melanin Synthesis and Tyrosinase Activity in B16F10 Cells

**Figure 1:**
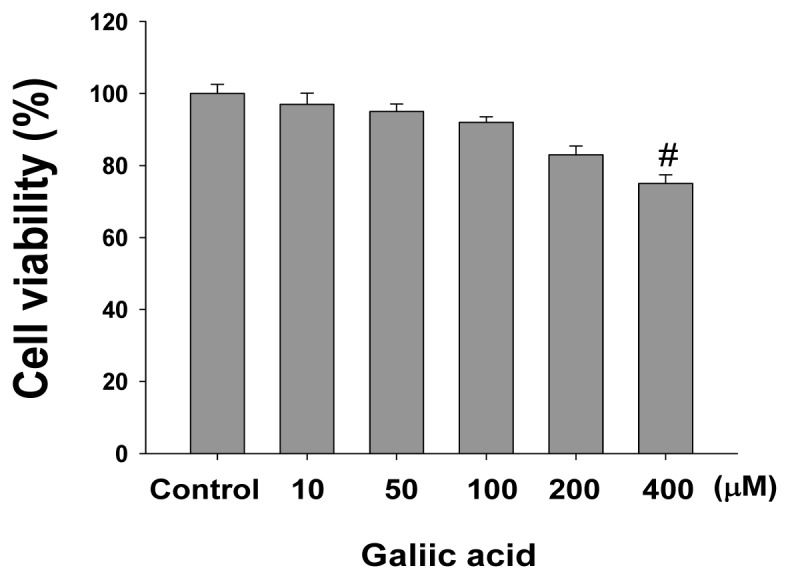
Effect of gallic acid on the cell viability of B16F10 cells. B16F10 melanoma cells were treated with various concentrations of gallic acid for 24 h, and cell viability was determined by 3-(4,5-cimethylthiazol-2-yl)-2,5-diphenyl tetrazolium bromide (MTT) assay. The data presented are from three independent experiments (^#^*p* < 0.05 compared with the control).

**Figure 2:**
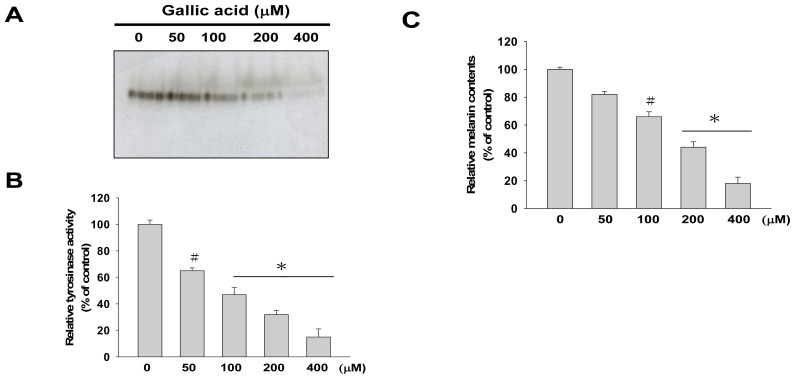
Tyrosinase activity and melanin synthesis in B16F10 cells with gallic acid treatment. Cells were treated with 0–400 μM gallic acid to evaluate the cellular tyrosinase activity and cellular melanin content. (**A**) Cellular tyrosinase activity stain; (**B**) Cellular tyrosinase activity assay; (**C**) Cellular melanin content. The data presented are from three independent experiments (^#^*p* < 0.05, * *p* < 0.001 compared with the control).

The cytotoxic effect of gallic acid was examined in mouse melanocyte cells, B16F10. After treatment with gallic acid at various concentrations (0, 10, 50, 100, 200, 400 μM) for 24 h, cell viabilities were determined by MTT assay. The results indicated that gallic acid was slightly cytotoxic to B16F10 cells at a concentration higher than 200 μM (Figure 1). Therefore, to investigate the effects of gallic acid on melanin synthesis and tyrosinase activity, B16F10 cells were then treated with 0, 50, 100, and 200 μM of gallic acid. As shown in Figure 2, tyrosinase activity staining, tyrosinase activity and cellular melanin contents were dose-dependently decreased by exposure to gallic acid. These results suggested that gallic acid has inhibitory effects on melanin synthesis through regulating tyrosinase and subsequently inhibiting melanin synthesis in B16F10 cells.

### 2.2. Effects of Gallic Acid on Expressions of Melanogenesis-Related Proteins

**Figure 3:**
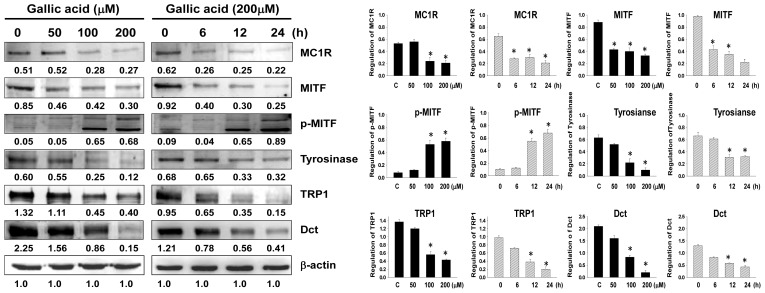
Expressions of melanogenesis-related proteins in B16F10 cells with gallic acid treatment. Western blotting data show the changes in MC1R, MITF, p-MITF, tyrosinase, TRP1, and Dct expressions in B16F10 melanoma cells treated with gallic acid at different concentrations (0–200 μM) for 24 h and treated with 200 μM of gallic acid at different times. β-Actin was used as the protein loading control. Statistical results represented as Means ± SEM (*n* = 3) by ANOVA with the Tukey-Kramer test (* *p* < 0.001 compared with the control).

To investigate whether gallic acid affects the expressions of melanogenesis-related proteins, including MC1R, MITF, p-MITF, CREB, p-CREB, tyrosinase, TRP-1, and Dct, these protein levels were examined in B16F10 cells using western blot analysis after treatment with different concentrations of gallic acid (0, 50, 100 and 200 μM). The expressions of melanogenesis-related proteins MC1R, MITF, p-CREB, tyrosinase, TRP-1, and Dct were dose- and time-dependently down-regulated after treatment with gallic acid in B16F10 cells (Figure 3). The results indicated that the suppressive activity of gallic acid on melanogenesis is linked to the down-regulation of MITF and other melanogenesis-related proteins.

### 2.3. Effect of Gallic Acid on the Melanogenesis-Related Signaling Pathway

**Figure 4:**
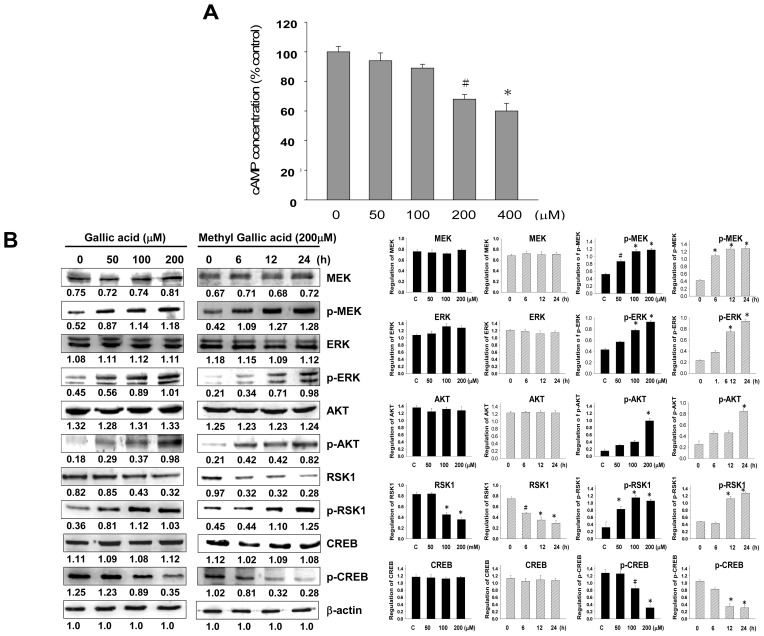
Intracellular cAMP concentration and the expressions of melanogenesis-related proteins in B16F10 melanoma cells with gallic acid treatment. (**A**) Cells were treated with 0~400 μM gallic acid for the indicated durations. Intracellular cAMP levels were measured using a cAMP ELISA kit. The results were representative of three independent experiments (^#^*p* < 0.05, * *p* < 0.001 compared with the control); (**B**) The western blot assay results showed the changes in p-MEK, MEK, p-ERK, ERK, p-Akt, Akt, p-RSK1, RSK1, p-CREB, and CREB expressions in B16F10 melanoma cells treated with different concentrations of gallic acid (0–200 μM) for 24 h and treated with 200 μM gallic acid at different times. β-Actin was used as the protein loading control. Statistical results represented as Means ± SEM (*n* = 3) by ANOVA with the Tukey-Kramer test (^#^*p* < 0.05, * *p* < 0.001 compared with the control).

We found that the inhibitory effect of gallic acid was exhibited through MITF down-regulation, which consequently inhibited the expressions of tyrosinase and melanogenesis-related proteins. In melanogenesis, MITF is regulated through the cAMP-mediated pathway by CREB phosphorylation, which is found to up-regulate MITF transcription [25]. We analyzed the intracellular cAMP levels after gallic acid treatment, and found that the intracellular cAMP levels were down-regulated in a dose-dependent manner after gallic acid treatment (Figure 4A). These results indicate that signal transduction could be hindered by gallic acid through the inhibition of intracellular cAMP. To further investigate the relationship between gallic acid and the cAMP-related signaling pathway, western blot analysis was used to assess MEK, p-MEK, ERK, p-ERK, Akt, p-Akt, RSK1, and p-RSK1. p-MEK, p-ERK, p-Akt, and p-RSK1 were significantly increased after gallic acid treatment. The phosphorylation level of CREB was down-regulated after gallic acid treatment, but CREB was unchanged (Figure 4B). These results suggest that the inhibitory effect of gallic acid may be related to CREB phosphorylation and activation by PI3K/Akt and MEK/ERK phosphorylation, indicating that the inhibitory effect of gallic acid on melanogenesis is related to the activation of phosphorylation of PI3K/Akt and MEK/ERK.

### 2.4. Effects of PI3K/Akt and MEK/ERK Inhibitors on Melanin Synthesis in Gallic Acid-Treated B16F10 Melanoma Cells

**Figure 5:**
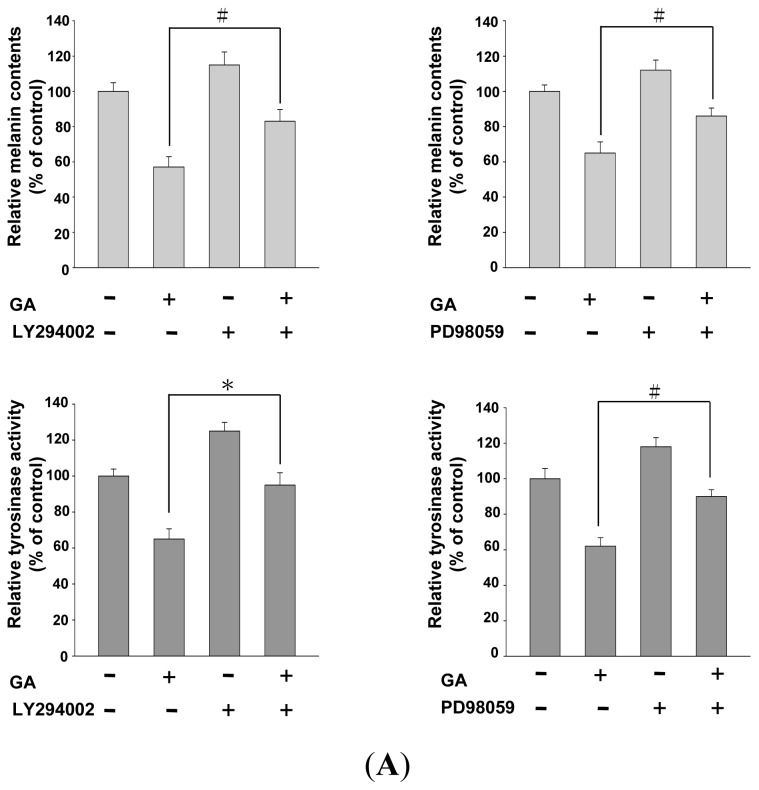

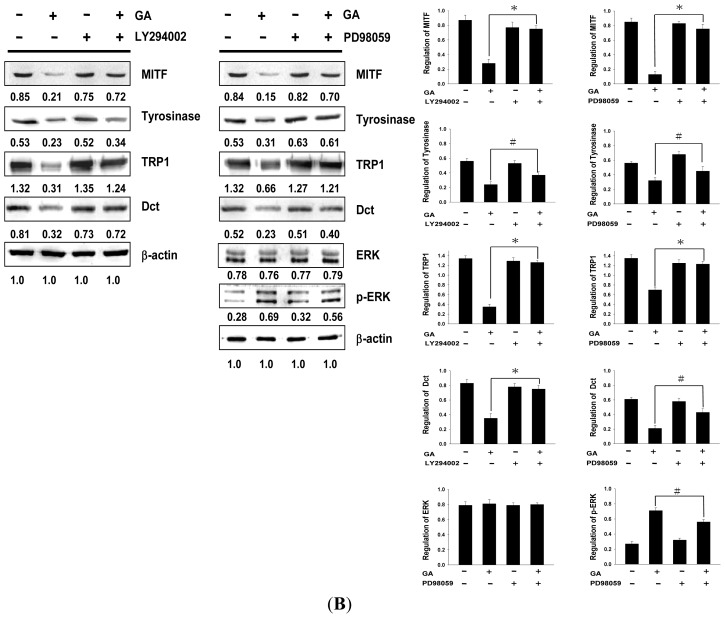
Recovery effect of co-treatment with PI3K/Akt or MEK/ERK specific inhibitors and gallic acid on cellular melanin content, cellular tyrosinase activity and melanogenesis-related proteins expression in B16F10 cells. Cells were pretreated with (or without) 20 μM MEK/ERK inhibitor (PD98059) or PI3K/Akt inhibitor (LY294002) for 1 h and further incubated with 200 μM gallic acid for 24 h. (**A**) The cellular melanin content and cellular tyrosinase activity were evaluated. The results shown are representative of three independent experiments (^#^*p* < 0.05, * *p* < 0.001 compared with the control); (**B**) Melanogenesis-related proteins expressions were analyzed by western blotting. β-Actin was used as the protein loading control. (GA: gallic acid). Statistical results represented as Means ± SEM (*n* = 3) performed by ANOVA with the Tukey-Kramer test (^#^*p* < 0.05, * *p* < 0.001 compared with the control).

As gallic acid decreases the intracellular cAMP levels and reduces melanin synthesis by increasing the phosphorylation and activation of PI3K/Akt and MEK/ERK, we further investigated whether the effects of gallic acid on tyrosinase activity and melanin synthesis are responsible for MEK/ERK and PI3K/Akt phosphorylation. We studied the melanin content and tyrosinase activity in B16F10 cells co-treated with gallic acid and PI3K/Akt-specific inhibitor (LY294002) or MEK/ERK-specific inhibitor (PD98059). Both, LY294002 and PD98059 treatment, restored melanin synthesis, whereas tyrosinase activity was suppressed by gallic acid in B16F10 cells (Figure 5A). These results suggest that gallic acid-induced PI3K/Akt and MEK/ERK phosphorylation reduce tyrosinase and melanogenesis-related protein expression involving MITF and appear to suppress melanin synthesis in B16F10 cells (Figure 5B). The results suggest that gallic acid induced PI3K/Akt and MEK/ERK phosphorylation, thus, down-regulating tyrosinase, MITF, TRP1, and Dct expression levels, and resulted in decreasing melanin synthesis in B16F10 cells.

### 2.5. Effect of Gallic Acid on Wnt/β-Catenin Signaling Pathway

**Figure 6:**
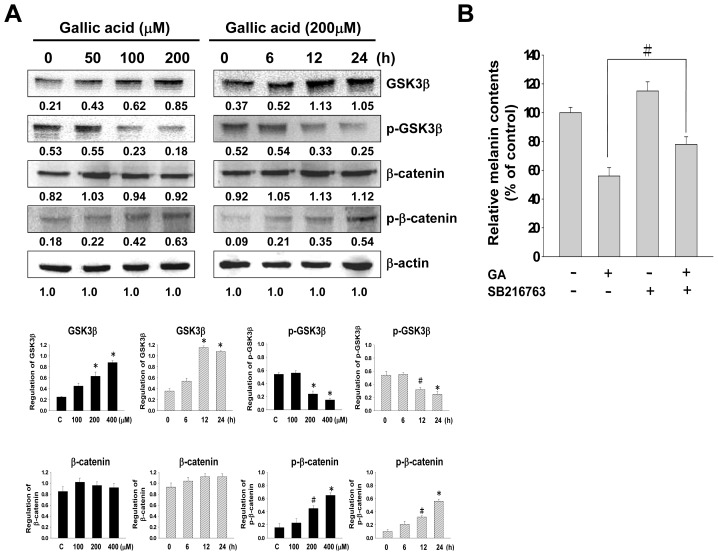
The expressions of Wnt/β-catenin related-proteins in B16F10 melanoma cells treated with gallic acid. (**A**) The Western blot assay results showed the changes in GSK3β, p-GSK3β, β-catenin, and p-β-catenin expressions in B16F10 melanoma cells treated with different concentrations of gallic acid (0–200 μM) for 24 h and treated with 200 μM of gallic acid at different times. β-Actin was used as the protein loading control. Statistical results represented as Means ± SEM (*n* = 3) performed by ANOVA with the Tukey-Kramer test (^#^*p* < 0.05, * *p* < 0.001 compared with the control); (**B**) The cellular melanin content was evaluated. These data are representative of three independent experiments (^#^*p* < 0.05 compared with the control).

As gallic acid increases the phosphorylation and activation of PI3K/Akt and MEK/ERK, we further investigated whether the effects of gallic acid on Wnt/β-catenin signaling pathway. GSK3β may contribute to maintaining the levels of functional MITF in melanocytes. The increasing level of cAMP activates GSK3β and increases ability of MITF to bind with its target sequences [26]. In our results, gallic acid inhibited intracellular cAMP. To further investigate the relationship between gallic acid and the Wnt/β-catenin signaling pathway, western blot analysis was used to evaluate the protein expression of GSK3β, p-GSK3β, β-catenin, and p-β-catenin. After treating with gallic acid, the phosphorylation level of GSK3β was down-regulated, but GSK3β and p-β-catenin were up-regulated (Figure 6A). We also studied the melanin content in B16F10 cells co-treated with gallic acid and GSK3β specific inhibitor (SB216763). SB216763 treatment restored gallic acid-induced melanin reduction (Figure 6B).

## 3. Discussion

Gallic acid is a plant polyphenol antioxidant that has been shown to have various biological properties. In the current study, we investigated the molecular mechanism of the inhibitory effect of gallic acid on melanogenesis in B16F10 cells. We firstly evaluated whether gallic acid has potential cytotoxicity to B16F10 cells, and found that gallic acid had no significant cytotoxic effect at 0–200 μM to the cells. Next, we found that gallic acid significantly suppressed melanin synthesis and tyrosinase in a dose-dependent manner. These results suggest that gallic acid decreases cellular melanin synthesis in B16F10 cells through down-regulating the tyrosinase activity. To understand the molecular mechanisms of the gallic acid-induced inhibitory effect, we examined the effect of gallic acid on melanogenesis-related proteins expressions, and MITF, tyrosinase, TRP1, and Dct were significantly dose- and time-dependently decreased after gallic acid treatment. These results suggest that gallic acid inhibits tyrosinase activity indirectly, through down-regulation of MITF and other melanogenesis-related proteins in B16F10 cells.

Although melanogenesis-related proteins execute critical roles in melanin synthesis, melanogenesis regulation also involves other non-enzymatic reactions. A recent study reported that degradation of MITF is related to the suppression of the α-MSH-induced cAMP-dependent melanogenic pathway in melanoma cells [27]. Consequently, we theorized that gallic acid-induced MITF down-regulation is also involved in the cAMP signal transduction cascade. As expected, gallic acid showed a dose-dependent inhibition effect on the intracellular cAMP levels (Figure 4A). These results suggest that the inhibitory effect of gallic acid is carried out by controlling melanogenesis-related proteins expressions through suppressing the crucial production of cAMP.

MITF plays a central role in melanin synthesis, as well as melanosome biogenesis and transport [28,29]. MITF also up-regulates the transcription of melanogenic proteins, including tyrosinase, TRP-1, Dct, and MC1R, and therefore it consequently induces melanin synthesis [29,30]. As the MEK/ERK-dependent pathway is responsible for regulation of MITF activity and stability through its phosphorylation, phosphorylation of MITF decreases its stability and thus leads to its degradation in proteosomes, which in turn reduces melanin synthesis [8,31]. Moreover, melanogenesis is also decreased by the phosphorylation of MITF on serine 29, which activates the PI3K/Akt signaling pathway [32]. The regulation of melanogenesis is associated with activation of the MEK/ERK and PI3K/Akt signaling pathways [33]. In particular, cAMP signaling is mediated via a PI3K-dependent mechanism that involves both the PKA and Ras/ERK signaling pathways in melanogenesis [25]. In human melanoma cells, either the elevation of the cAMP level or inhibition of PI3K expression increase the melanin content by diminishing Akt phosphorylation [26]. Furthermore, PKA is involved in the phosphorylation of CREB, and activation of CREB phosphorylation has been found to induce MITF transcription. Therefore, we further investigated whether gallic acid is involved in the regulation of downstream cAMP signaling. Interestingly, our results showed that in concordance with the dose-dependent effect on cAMP suppression, gallic acid treatment up-regulated PI3K/Akt and MEK/ERK phosphorylation in a dose- and time-dependent manner, while gallic acid treatment down-regulated CREB phosphorylation.

In order to ensure that these signaling factors are responsible for the gallic acid-induced inhibitory effects on melanogenesis, we blocked PI3K/Akt or MEK/ERK signaling in the presence of gallic acid and evaluated melanin production and tyrosinase activity in B16F10 cells. Our results demonstrated that the specific PI3K inhibitor, LY294002, significantly retrieved melanin production and tyrosinase activity, and the MEK/ERK inhibitor, PD98059, markedly inhibited the gallic acid suppressive effects on MITF, tyrosinase, TRP-1, and Dct expression. The findings support that gallic acid may inhibit the signaling pathways related to melanogenesis, which include the inhibition of MITF-tyrosinase signaling, in particular the activation of PI3K/Akt or MEK/ERK signaling. The recent report showed that gallic acid suppressed melanogenesis in melanoma cells through inhibition of tyrosinase, TRP-1, Dct, and MITF. Gallic acid may activate Akt and ERK signaling, which induce proteasomal degradation of MITF. Their results also obtained from *in vivo* experiments showed that gallic acid potentially ameliorates and reversed UVB-induced hyperpigmentation and zebrafish body pigmentation [34]. These results agree with our findings.

In addition, we investigated the possible role of Wnt/β-catenin signaling pathway in melanin synthesis. Our results showed that gallic acid treatment up-regulated GSK-3β and p-β-catenin in a dose- and time-dependent manner, while gallic acid treatment down-regulated p-GSK-3β. GSK-3β, a negative regulator in the Wnt/β-catenin signaling pathway, could activate the function of MITF through phsphorlyation at Ser 298 [35]. Activated GSK-3β induces phosphorylation of the *N*-terminal Ser/Thr residues in β-catenin, which leads to the ubiquitination and degradation of β-catenin [16]. We investigated further for the possible involvement of GSK-3β in melanognesis using SB216763, a GSK-3β specific inhibitor. SB216763 recovered gallic acid-induced melanin reduction (Figure 6B). The regulation of melanin synthesis results from cross-talk between several different signaling pathways. These results indicate that the inhibitory effects of gallic acid against melanogenesis are associated with the activation of PI3K/Akt or MEK/ERK signaling pathway, the inactivation of Wnt/β-catenin and cAMP signaling pathway.

## 4. Experimental

### 4.1. Chemicals and Reagents

Protease inhibitor cocktail, gallic acid, melanin, 3,4-Dihydroxyphenylalanine (l-DOPA), MTT, PD98059, LY294002, SB216763, and goat anti-rabbit β-Actin antibody were purchased from Sigma-Aldrich (St. Louis, MO, USA). PVDF (polyvinylidene difluoride) membranes were obtained from Millipore (Bellerica, MA, USA). Chemiluminescent HRP substrate was purchased from Pierce (Rockford, IL, USA). Antibodies against TRP1, Dct, ERK, and p-ERK were obtained from ProteinTech Group (Chicago, IL, USA). Antibodies against MITF, p-MITF, MEK, and p-MEK were purchased from Assay BioTech (San Francisco, CA, USA). Antibodies against tyrosinase, CREB, p-CREB, MC1R, Akt, p-Akt, ASK1, and p-ASK1 were purchased from Epitomics (Burlingame, CA, USA). Antibodies against GSK3β, p-GSK3β, β-catenin, and p-β-catenin were purchased from Cell Signaling Technology (Danvers, MA, USA). cAMP ELISA kit was purchased from Enzo Life Sciences (Farmingdale, NY, USA).

### 4.2. Cell Culture and Treatment with Gallic Acid

B16F10 mouse melanoma cells were cultured in Dulbecco’s modified Eagle’s medium (DMEM; Gibco Life Technologies, Carlsbad, CA, USA) with 4 mM l-glutamine, 1.5 g/L sodium bicarbonate, 10% FBS, 100 U/mL penicillin, and 100 μg/mL streptomycin at 37 °C in a humidified 5% CO_2_ atmosphere. Gallic acid dissolved in DMSO. Cells were cultured with different concentrations of gallic acid (50, 100, 200 and 400 μM) and harvested after 24 h of incubation. B16F10 cell control cultures were made by adding DMSO (0.01% *v*/*v*) at the same final concentration as in the gallic acid-treated samples. Experiments were performed in triplicate and repeated multiple times.

### 4.3. Cell Viability Assay

The effect of gallic acid against B16F10 cells was determined using a cell viability assay through the reduction of 3-(4,5-cimethylthiazol-2-yl)-2,5-diphenyl tetrazolium bromide (MTT) to formazan. B16F10 cells (1 × 10^4^ cells/well) were incubated in 96-well plates. After treatment with various concentrations of gallic acid, the cells were incubated at 37 °C for 24 h. MTT solution (1 mg/mL, 50 μL/well) was then added to each well, and the plates were incubated at 37 °C for 4 h. The resulting crystals were solubilized in DMSO, and the absorbance was measured using a microtiter plate ELISA reader at 595 nm [36].

### 4.4. Cellular Melanin Content Determination

The melanin content was measured according to a previously described method [37]. B16F10 cells (1 × 10^5^ cells/well) were incubated in 24-well plates. Briefly, B16F10 cells were treated with gallic acid for 24 h. The cells were washed with PBS and then dissolved in 200 μL of 1 N NaOH at 80 °C for 2 h. The samples were centrifuged for 30 min at 12,000 rpm to collect the supernatant. The relative melanin content was determined by measuring the absorbance at 475 nm on a microtiter plate ELISA reader. Standard curves using synthetic melanin (0–300 μg/mL) were prepared for each experiment. Melanin production was calculated as the μg of melanin/μg of total proteins in a cell extract.

### 4.5. Tyrosinase Activity Assay

Tyrosinase activity was estimated by measuring the rate of L-DOPA oxidation using a method described previously [38]. B16F10 cells (1 × 10^5^ cells/well) were incubated in 24-well plates. Briefly, the cells were lysed with cell lysis buffer (50 mM Tris-HCl (pH 7.0), 2% β-mercaptoethanol, 10 mM EDTA and protease inhibitor cocktail). The cell lysates were then centrifuged at 12,000 rpm for 30 min at 4 °C to collect the supernatant. The supernatant was then tested for the cell tyrosinase activity. The reaction mixture contained 0.1 M sodium phosphate (pH 7.0) reacted with an equal volume of 1 mg/mL l-DOPA. After incubation at 37 °C for 2 h, the dopachrome was observed by measuring the absorbance at 405 nm in an ELISA reader. Each measured result was expressed as the percentage change from the control.

### 4.6. Tyrosinase Activity Staining

The tyrosinase activity staining was analyzed according to a previously described method [39]. B16F10 cells (5 × 10^6^ cells/well) were incubated in 10-cm dish plates. Briefly, the cells were lysed with cell lysis buffer (50 mM Tris-HCl (pH 7.0), 2% β-mercaptoethanol, 10 mM EDTA and protease inhibitor cocktail). The protein concentrations were determined by a Bradford Assay (Bio-Rad, Hercules, CA, USA), after which 25 μg of total protein were separated by 10% SDS-PAGE. The sample was prepared in 0.1% SDS and β-mercaptoethanol and heat treatment was avoided. After electrophoresis, the gels were soaked in 10 mM Na_2_HPO_4_ buffer (pH 6.2) for 30 min, followed by incubation in the same buffer containing 2 mM l-DOPA at 37 °C.

### 4.7. Western Blot Analysis

Western blot analysis was performed according to a previously described method [40]. B16F10 cells (5 × 10^6^ cells/well) were incubated in 10-cm dish plates. Briefly, the cells were lysed with cell lysis buffer (50 mM Tris-HCl (pH 7.0), 2% β-mercaptoethanol, 10 mM EDTA and protease containing protease inhibitor cocktail. Proteins (25 μg) extracted from whole cells were separated by 12.5% SDS gel electrophoresis and then transferred onto a PVDF membrane (Millipore) for 2 h at 400 mA using Transphor TE 62 (Hoeffer). The PVDF membranes were then incubated with appropriate rabbit polyclonal antibodies against mouse MITF, p-MITF, tyrosinase, TRP-1, Dct, CREB, p-CREB, ERK, p-ERK, MEK, p-MEK, RSK1, p-RSK1, MC1R, GSK3β, p-GSK3β, β-catenin, p-β-catenin, and β-actin at 4 °C for 2 h or overnight. The membranes were washed three times in PBST (PBS buffer containing 0.05% Tween 20) and then probed with goat anti-rabbit horseradish peroxidase conjugated antibody (1:5000) for 1 h. The blots were then visualized using ECL Western Blotting Reagents (Pierce, Rockford, IL, USA). The Western blot data were quantified with Image J 1.47 software (http://www.downloadcrew.com/article/28008-imagej).

### 4.8. cAMP Assay

cAMP assay was performed with cell lysate using a cAMP assay kit (Enzo Life Sciences, Farmingdale, NY, USA) in accordance with the manufacturer’s protocol. Brief, B16F10 cells were seeded on 96-well plates at a density of 1 × 10^4^ cells/well. Cells were treatment with gallic acid at various concentrations (0, 50, 100, 200, 400 μM), and were lysed using cell lysis buffer. Lysates were centrifuged at 3000 rpm at 4 °C, and then supernatant was used directly. The cell lysates supernatant were added to the specific cAMP polyclonal antibody-coated plates afterwards. Incubation was performed with antibody for 1 h at room temperature, and the excess monoclonal antibody was removed by washing buffer for three times. After the process, standard and cAMP-HRP conjugate were added to wells and incubated for 2 h on a shaker at room temperature and the washing procedure was repeated. The concentration of cAMP was observed by measuring the absorbance at 450 nm in an ELISA reader.

## 5. Conclusions

In conclusion, we investigated the effects of gallic acid on the molecular mechanism of the inhibition of melanin production and tyrosinase activity. In this study, gallic acid was found to inhibit melanogenesis in B16F10 cells by inhibiting the expressions of MITF, tyrosinase, TRP1, and Dct, which are mediated by PI3K/Akt or MEK/ERK phosphorylation. Furthermore, the inhibitory effect of gallic acid may be involved in the suppression of intracellular cAMP production and inhibition of Wnt/β-catenin pathway. These data indicate the possible mechanisms of inhibitory effects of gallic acid against melanogenesis may contain the decrease in cAMP as well as PI3K/Akt and MEK/ERK phosphorylation. Our study provides new insight into the role that gallic acid plays in melanogenesis. Therefore, gallic acid may be a potentially useful therapeutic agent for use in reducing skin hyperpigmentation and may be an effective component in lightening and whitening cosmetics.
